# The Prevalence of and Factors Associated with Current Smoking among College of Health Sciences Students, Mekelle University in Northern Ethiopia

**DOI:** 10.1371/journal.pone.0111033

**Published:** 2014-10-23

**Authors:** Tadele Eticha, Feven Kidane

**Affiliations:** Department of Pharmacy, College of Health Sciences, Mekelle University, Mekelle, Ethiopia; Lovelace Respiratory Research Institute, United States of America

## Abstract

**Background:**

Tobacco smoking is one of the greatest causes of preventable morbidity and mortality globally, and is responsible for many causes of untimely deaths. This survey was aimed to determine prevalence and factors associated with current smoking among the students of College of Health Sciences, Mekelle University, Ethiopia.

**Methods:**

A cross-sectional study was employed using a structured self-administered questionnaire among College of Health Sciences students in March 2013. A stratified random sampling method was employed to select study participants. Data were entered and analysed using of Statistical Package for Social Sciences (SPSS) version 20.0.

**Results:**

Of the 193 students, 57 (29.5%) of the students were current smokers. Most of the current smokers (89.4%) smoked between 1–10 sticks of cigarette per day. The two main reasons cited for smoking cigarettes were peer pressure (43.9%) and to relieve stress (36.8%). Being female (adjusted OR [AOR] = 0.49; 95% CI: 0.25, 0.95) and Tigre by ethnicity (AOR = 0.32; 95% CI: 0.14, 0.74) were significantly less associated with current smoking. On the other hand, being second year students (AOR = 3.84; 95% CI: 1.41, 10.46), khat chewing (AOR = 8.36; 95% CI: 2.60, 26.85) and taking illicit drugs (AOR = 10.59; 95% CI: 2.77, 40.51) were positively associated with current smoking cigarettes.

**Conclusions:**

The current smoking prevalence among students in College of Health Sciences, Mekelle University is high and therefore, effective smoking prevention and cessation intervention programs are required to reduce smoking among university students.

## Introduction

Tobacco smoking is one of the greatest causes of preventable morbidity and mortality globally, and is responsible for many causes of untimely deaths [1]. Cigarette smoking causes lung cancer, chronic obstructive lung disease, atherosclerotic cardiovascular diseases, peptic ulcer disease, intrauterine growth retardation, spontaneous abortion, antepartum hemorrhage, female infertility, sexual dysfunction in men, and many other diseases [2]. Globally, it has become a rapidly growing problem of public health concern as it has been calculated that nearly a third of the world’s population, aged 15 years above, are smokers [3]. The prevalence is on the rise, especially in developing countries [1].

College life is an important transition period during which young adults set out to explore tobacco use [4]. Some surveys have reported that the prevalence of cigarette smoking continues to rise among college students [5], [6]. In sub-Saharan Africa, national smoking prevalence among men varies from 20% to 60% and the annual cigarette consumption rates are placed on the rise of both genders [7]. A study conducted among college students in northwest Ethiopia revealed 13.1% lifetime prevalence rate of cigarette smoking and 8.1% current prevalence of cigarette smoking [8]. Another survey among undergraduate medical students at Addis Ababa University reported a lifetime smoking prevalence of 9% and the current smoking prevalence of 1.8% [9]. A survey conducted among university students in southwest Nigeria showed that the prevalence of ever smoked was 22.0%, while those that currently smoke were 13.7% [10]. Similarly, a study conducted among university students in Cameroon reported an ever smoking prevalence of 30.1% and the current smoking prevalence of 6.3% [11].

An increasing trend is anticipated to occur among university students and this could be related to alleviation of stress, life problems, peer pressure, social acceptance, class history of smoking, lower educational level of parents and the desire to attain high personality profile [12]. There is little data regarding the prevalence of smoking and its predictors among university students in Ethiopia though some studies conducted among school adolescents. Thus, the purpose of the present work was to determine the prevalence of current smoking and its related factors among College of Health Sciences (CHS) students, Mekelle University.

## Methods

### Settings and study design

A cross-sectional study was undertaken in College of Health Sciences, Mekelle University found in Mekelle town in March 2013. Mekelle, the capital city of the Tigray Regional State, is located 780 km north of Addis Ababa, which is the capital city of Ethiopia. Mekelle University is one of the 23 public funded universities in Ethiopia. The CHS consists of the School of Medicine and departments such as Pharmacy, Public Health, Nursing and Midwifery.

### Study participants and sampling

Regular students, who were currently studying a bachelor level in any discipline, were eligible for the study. The sample size was calculated by using the formula for a single population proportion by considering 95% confidence interval, 5% margin of error, 13.1% of the prevalence rate of cigarette smoking among college students in northwest Ethiopia [8] and 10% of non-response rate. The calculated sample size was 175 and adding a 10% of the non-response rate to get a total sample of 193.

A stratified random sampling technique was used to collect the data. Stratification was done based on the year of study and discipline to select study participants. The study was approved for ethical issues by the Health Research Ethics Review Committee of College of Health Sciences, Mekelle University. The students were briefed on the aims and objectives of the study and written informed consent was obtained from those who were willing to participate in the study.

The following operative definitions were used: never smoker was a student who had never tried a cigarette in his/her lifetime; former smoker was a student who ever smoked, but had stopped smoking at the time of assessment while currently smoker was a student who smoked cigarette one or more in the past 30 days prior to the survey. Ever smokers included current and former smokers, and non-smoker included never and former smokers. Khat is a perennial shrub whose fresh leaves and soft twigs are chewed for its stimulant effect.

### Data collection and analysis

Data were collected by a structured self-administered questionnaire adopted from other similar setups [13], [14]. To ensure quality of the data, pre-test was conducted at 5% of the sample size in similar setups before the actual data collection. The questionnaire consisted of demographic characteristics and smoking behavior.

Data were entered and analyzed using of Statistical Package for Social Sciences (SPSS) version 20.0. Descriptive statistics and logistic regression were performed. Multivariate logistic regression analysis was used to identify factors independently associated with the current smoking status of university students. In this model, variables with a bivariate test value ≤ 0.05 were included. All p values were two tailed with the significance level set at 0.05.

## Results

### Sociodemographic characteristics

There were a total number of 193 respondents included in the present work. About half (49.7%) of the students were male. The age of respondents ranged from 18 to 28 years with a mean age of 21.2±1.7 SD years. The predominant religion of respondents was Orthodox constituting 86.5% of total respondents. The majority (64.8%) of the students were Tigre by ethnicity and 63.2% of the students getting greater than 500 Ethiopian Birr (ETB) (exchange rate 1 USD = 18.8 ETB) monthly from their families. Just above one fourth (27.5%) of the study participants were second year students and 103(53.4%), 37(19.2%) and 53(27.5%) were taken from the School of Medicine, Departments of Pharmacy and other health sciences, respectively. Fathers of 13.0% and mothers of 21.8% of the respondents were illiterates whereas fathers of 32.1% and mothers of 30.1% of the respondents had primary education. On the other hand, fathers of 37.3% and mothers of 20.0% of the students had tertiary education.

### Prevalence of smoking and its associated factors

**Figure 1:**
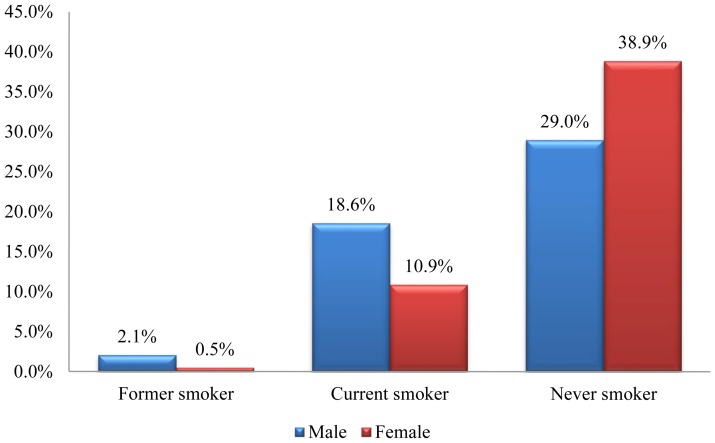
Prevalence of smoking among CHS Students, March 2013 (N = 193). Figure 1 indicates the prevalence of smoking among CHS students by gender. Out of the 193 respondents, 57 (29.5%) of the students were current smokers while 5 (2.6%) were former smokers.

Prevalence of smoking among CHS students by gender is shown in Figure 1. Out of the 193 respondents, 57 (29.5%) of the students were current smokers while 5 (2.6%) were former smokers. The prevalence of current smoking was 37.5% among males and 21.6% among females. Smoking was currently practiced by 30(29.1%) of medical students, 13(35.1%) of pharmacy students and 14(26.4%) of other health sciences students.

Sociodemographic and drug related factors associated with current smoking among CHS students are presented in Table 1 and Table 2. The bivariate analysis revealed that gender, ethnic group, year of study, fathers’ education, mothers’ education, having smoking friends, alcohol consumption, khat chewing, taking illicit drugs and smoking harmful were found to be significantly related to current smoking. The final multiple logistic regression model indicated that gender (AOR = 0.49; 95% CI: 0.25, 0.95), ethnic group (AOR = 0.32; 95% CI: 0.14, 0.74), second year students (AOR = 3.84; 95% CI: 1.41, 10.46), khat chewing (AOR = 8.36; 95% CI: 2.60, 26.85) and taking illicit drugs (AOR = 10.59; 95% CI: 2.77, 40.51) were independent factors associated with the current smoking status of university students. However, fathers’ education, mothers’ education, having smoking friends, alcohol consumption and smoking harmful were retained in the multivariate model as confounders of independent predictors of current cigarette smoking.

**Table 1:** Sociodemographic factors associated with current smoking among CHS Students, March 2013.

Characteristics		Smoking status,n(%)		COR(CI 95%)	AOR^¶^(CI 95%)	P value
		Smoker	Non-smoker			
Gender	Male	36(37.5)	60(62.5)	1	1	
	Female	21(21.6)	76(78.4)	**0.46(0.24, 0.87)**	**0.49(0.25, 0.95)**	**0.035**
Age	15–19	8(21.6)	29(78.4)	0.60(0.26, 1.41)	–	
	≥20	49(31.4)	107(68.6)	1		
Religion	Orthodox	46(27.7)	120(72.3)	1	–	
	Other	11(40.7)	16(59.3)	1.79(0.78, 4.15)		
Ethnic group	Amhara	17(41.5)	24(58.5)	1	1	
	Oromo	6(50.0)	6(50.0)	1.41(0.39, 5.13)	1.87(0.46, 7.60)	0.384
	Tigre	28(22.4)	97(7.6)	**0.41(0.19, 0.86)**	**0.32(0.14, 0.74)**	**0.008**
	Other	6(40.0)	9(60.0)	0.94(0.28, 3.14)	1.15(0.32, 4.17)	0.834
Year of study	First	10(25.0)	30(75.0)	1	1	
	Second	24(45.3)	29(54.7)	**2.48(1.01, 6.09)**	**3.84(1.41, 10.46)**	**0.008**
	Third	9(25.0)	27(75.0)	1.00(0.35, 2.83)	1.37(0.45, 4.15)	0.577
	Fourth	7(20.0)	28(80.0)	0.75(0.35, 2.83)	1.22(0.38, 3.97)	0.738
	Fifth	7(24.1)	22(75.9)	0.96(0.31, 2.90)	0.73(0.22, 2.40)	0.601
Monthly income (ETB)	<250	8(21.6)	29(78.4)	0.57(0.24, 1.45)	–	
	250–500	9(26.5)	25(73.5)	0.74(0.32, 1.73)		
	>500	40(32.8)	82(67.2)	1		

COR = Crude Odds Ratio, AOR = Adjusted OR, CI = Confidence Interval, Exchange rate: 1 USD = 18.8 Ethiopian Birr (ETB), **^¶^**Adjusted for gender, ethnic group and year of study.

**Table 2:** Parent and drug related factors associated with current smoking among CHS Students, March 2013.

Characteristics		Smoking status,n(%)		COR(CI 95%)	AOR¶(CI 95%)	P value
		Smoker	Non-smoker			
Father’s education	Illiterate	7(28.0)	18(72.0)	0.54(0.20, 1.47)	0.23(0.30, 2.30)	0.228
	Primary	9(14.5)	53(85.5)	**0.24(0.10, 0.56)**	0.27(0.06, 1.28)	0.100
	Secondary	11(32.4)	23(67.6)	0.67(0.28, 1.58)	0.34(0.85, 1.39)	0.133
	Tertiary	30(41.7)	42(58.3)	1	1	
Mother’s education	Illiterate	8(19.0)	34(81.0)	**0.34(0.13, 0.88)**	0.75(0.11, 5.31)	0.777
	Primary	12(20.7)	46(79.3)	**0.38(0.17, 0.88)**	1.18(0.26, 5.46)	0.829
	Secondary	15(38.5)	24(61.5)	0.91(0.39, 2.11)	2.26(0.60, 8.44)	0.227
	Tertiary	22(40.7)	32(59.3)	1	1	
Parent(s) smoking	Yes	13(40.6)	19(59.4)	1.82(0.83, 3.99)	–	
	No	44(27.3)	117(72.7)	1		
Friend(s) smoking	Yes	50(46.3)	58(53.7)	**9.61(4.06, 22.72)**	2.82(0.89, 8.96)	0.079
	No	7(8.2)	78(91.8)	1	1	
Alcohol consumption	Yes	46(52.3)	42(47.7)	**9.36(4.41, 19.85)**	2.62(0.97, 7.09)	0.058
	No	11(10.5)	94(89.5)	1	1	
Khat chewing	Yes	32(76.2)	10(23.8)	**16.13(7.04, 36.97)**	**8.36(2.60, 26.85)**	**0.000**
	No	25(16.6)	126(83.4)	1	1	
Taking illicit drugs	Yes	30(88.2)	4(11.8)	**36.67(11.94, 112.6)**	**10.59(2.77, 40.51)**	**0.001**
	No	27(17.0)	132(83.0)	1	1	
Smoking harmful	Yes	47(27.0)	127(73.0)	1	0.23(0.04, 1.21)	0.082
	No	10(52.6)	9(47.4)	**3.00(1.15, 7.85)**	1	

¶ Adjusted for mother’s education, friend smoking, alcohol consumption, khat chewing, taking illicit drugs and smoking harmful.

Nearly half (49.1%) of the current smokers started smoking after joining the university. Most of the current smokers (89.4%) smoked between 1–10 sticks of cigarette per day while just more than half (52.6%) of them smoked every day. The reasons mentioned for smoking cigarettes were peer pressure (43.9%), relieve stress (36.8%) and entertainment (12.3%). About 38.6% of smokers reported that they had tried to quit smoking previously but had failed. The majority (59.6%) of smokers intended to quit smoking (Table 3).

**Table 3:** Selected characteristics of current smokers in CHS, March 2013 (N = 57).

Characteristics		Frequency (%)
Started smoking since joining college	Yes	28(49.1)
	No	29(50.9)
Frequency of smoking	Every day	30(52.6)
	Few days in a week	13(22.8)
	Few days in a month	14(24.6)
Number of cigarette per day	1–10	51(89.4)
	>10	6(10.6)
Reasons for initiation	Peer pressure	25(43.9)
	Relieve stress	21(36.8)
	Entertainment	7(12.3)
	Availability of cigarette	4(7.0)
Tried to quit smoking in the past	Yes	22(38.6)
	No	35(61.4)
Willing to quit smoking	Yes	34(59.6)
	No	23(40.4)

## Discussion

This study looked into the prevalence and determinants of current cigarette smoking among CHS students, Mekelle University. The overall prevalence of current smoking among the respondents in our study was 29.5%, 37.5% among males and 21.6% among females. This prevalence is remarkably higher than the reports from other surveys conducted in different parts of Ethiopia in which the prevalence of current smoking ranged from 1.8% to 8.1% [8], [9], [13]–[17]. Likewise, lower prevalence rates of cigarette smoking have been reported in previous works [3], [10], [11], [18]. On the other hand, some studies conducted in other countries [19]–[22] have shown very high prevalence rates of cigarette smoking while other studies [23]–[31] have reported results that are comparable to our findings. The possible explanation for the observed differences in cigarette smoking could be due to lack of standard definitions for current cigarette smoking. Some studies define it in different ways though many studies define current smoking as having smoked at least once in the last 30 days prior to a survey conducted. The second reason for the differences in smoking might be as a result of small sample size employed in the present study. The third reason could also be due to the differences in awareness and knowledge of the health risks of smoking. The level of understanding, knowledge and awareness of the danger of cigarette smoking might be related to discipline. For instance, the study conducted among only medical students at Addis Ababa University reported low current smoking prevalence which shows that medical students have a better understanding of the harmful effects of smoking [9]. Furthermore, the heterogeneity of smoking prevalence among countries reflects differences in social and economic development [32].

It was discovered that khat chewing and taking illicit drugs were significantly associated with cigarette smoking in the present study. These are consistent with the study in which khat chewing was significantly associated with cigarette smoking [9]. Multiple substance use (such as smoking, khat and alcohol use) might be due to the need to increase satisfaction. In addition, different substance use could be used side by side to relieve stress. Although alcohol consumption was significantly associated with cigarette smoking in other studies [9], [10], it was found to be a confounding factor in this survey. Failure to detect an association between alcohol consumption and cigarette smoking in this survey might be on account of the low sample size.

This study also indicated a significantly higher prevalence rate of smoking among second year students. In contradiction to this, other studies showed that the higher number of years of university education was associated with increased prevalence of cigarette smoking [8], [27], [30]. About half of the current smokers started smoking after joining the university in the present study and hence the number of smokers was increased in the second year. However, the lower prevalence of smoking after the second year may be due to comparatively greater awareness and knowledge of the health risks of cigarette smoking in the upper level students.

Social and family situations can cause a great impact on whether someone decides to take up smoking. Young people who have family members or close friends who smoke are significantly more likely to smoke than those who don’t [10], [30], [31]. In some studies, family history of smoking was not associated with smoking though friend smoking habits were associated with smoking [11], [17], [19]. However, family history of smoking or having smoking friends was not significantly associated with current smoking in the present work. The small sample size used in this survey could affect the association between these factors and current smoking. On the other hand, it was ascertained that male sex was significantly associated with cigarette smoking in this study. Similar findings from other studies also described that there was a significantly higher prevalence of smoking among males, which might be as a consequence of the societal and cultural acceptance of the smoking habit among men rather than women [8], [10], [11], [19], [30]–[31].

Around half of the students began smoking after joining the university, a result which agrees with that reported in other studies [11], [31] which confirm that many students initiate smoking at university. The majority of current smokers reported that they started smoking due to peer pressure. This suggests that friends influence smoking behaviour and evidence from other studies shows that most people started smoking due to the influence of friends [8], [10], [21], [30], [31]. The ability to cope with stress was also one of the most common reasons given for smoking as reported in other studies [8], [21].

Most of the smokers consumed between 1–10 sticks of cigarette per day, which was similar to the findings of another study [21]. However, a study conducted among university students reported that most of the students smoked >10 sticks of cigarette per day [31]. Some smokers reported that they had attempted to stop smoking previously, but had failed like other studies [31], [33]. A high percentage of smokers intended to stop smoking, which was similar to the findings of a study among university students in Jordan [30]. The findings of our study indicate that a high percentage of current smokers may respond well to smoking cessation programs if these were made available to the university.

This study had several limitations. First, the survey used a cross-sectional study that cannot show direct causality of smoking, whether beneficial or harmful, among university students. Second, small sample size contributed to wider confidence intervals with low precision. Finally, the data were collected based on self-report of the students and may be subject to recall bias and under-reporting of cigarette smoking due to social desirability bias. In spite of these limitations, the findings suggest a demand to educate university students regarding cigarette smoking and its effects.

In conclusion, this survey indicated a high prevalence of current smoking cigarettes among CHS students, which can constitute a major public health problem in the future. This survey also identified that current smokers intended to quit smoking cigarettes. The findings of this study suggest that effective smoking prevention and cessation intervention programs are required in order to reduce smoking among university students.
